# The antibacterial effect of nitric oxide against ESBL-producing uropathogenic *E. coli* is improved by combination with miconazole and polymyxin B nonapeptide

**DOI:** 10.1186/1471-2180-14-65

**Published:** 2014-03-14

**Authors:** Charlotte Sahlberg Bang, Annica Kinnunen, Marie Karlsson, Anna Önnberg, Bo Söderquist, Katarina Persson

**Affiliations:** 1 Faculty of Medicine and Health, iRiSC - Inflammatory Response and Infection Susceptibility Centre, Örebro University, SE- 701 82 Örebro, Sweden; 2 Department of Laboratory Medicine, Clinical Microbiology, Örebro University Hospital, SE-701 85 Örebro, Sweden; 3 School of Medicine, Campus USÖ, Örebro University, SE-701 82 Örebro, Sweden

**Keywords:** Uropathogenic *E. coli*, Extended-spectrum β-lactamase, Nitric oxide, Polymyxin B nonapeptide

## Abstract

**Background:**

Nitric oxide (NO) is produced as part of the host immune response to bacterial infections, including urinary tract infections. The enzyme flavohemoglobin, coded by the *hmp* gene, is involved in protecting bacterial cells from the toxic effects of NO and represents a potentially interesting target for development of novel treatment concepts against resistant uropathogenic bacteria. The aim of the present study was to investigate if the *in vitro* antibacterial effects of NO can be enhanced by pharmacological modulation of the enzyme flavohemoglobin.

**Results:**

Four clinical isolates of multidrug-resistant extended-spectrum β-lactamase (ESBL)-producing uropathogenic *E. coli* were included in the study. It was shown that the NO-donor substance DETA/NO, but not inactivated DETA/NO, caused an initial growth inhibition with regrowth noted after 8 h of exposure. An *hmp*-deficient strain showed a prolonged growth inhibition in response to DETA/NO compared to the wild type. The imidazole antibiotic miconazole, that has been shown to inhibit bacterial flavohemoglobin activity, prolonged the DETA/NO-evoked growth inhibition. When miconazole was combined with polymyxin B nonapeptide (PMBN), in order to increase the bacterial wall permeability, DETA/NO caused a prolonged bacteriostatic response that lasted for up to 24 h.

**Conclusion:**

An NO-donor in combination with miconazole and PMBN showed enhanced antimicrobial effects and proved effective against multidrug-resistant ESBL-producing uropathogenic *E. coli*.

## Background

Nitric oxide (NO) is formed from an inducible form of nitric oxide synthase (iNOS) as part of the host antimicrobial defence program [1]. Increased iNOS activity has been documented in the bladder and urine in patients suffering from urinary tract infection (UTI) [2,3]. The antimicrobial effects of NO are complex but NO and its autoxidation products interact with a wide variety of targets including iron-sulfur thiols, tyrosine residues, lipids and DNA bases [1,4]. NO inhibits growth of a wide variety of gram-negative and gram-positive bacterial species [1]. Growth inhibitory effects on common urinary pathogens have been reported [5], but uropathogenic *E. coli* (UPEC) are less sensitive to NO than non-pathogenic strains of *E. coli*[6,7].

To protect against the damage caused by nitrosative stress, bacteria possess multiple defence mechanisms involving scavengers, detoxifying enzymes and evasion strategies. Flavohemoglobin, encoded by the *hmp* gene [8], is a protein that has been shown to have NO dioxygenase activity [8] and be involved in protecting bacterial cells from nitrosative stress [9,10]. Flavohemoglobin utilizes O_2_ for NO detoxification and oxidize NO to harmless nitrate, which protect the bacteria from the toxic effect of NO. The significance of flavohemoglobin in NO-protection has been shown using *hmp*-deficient mutants that are more sensitive to NO and nitrosative stress [8,11]. We have previously shown that UPEC increase flavohemoglobin expression after exposure to NO and that an *hmp*-deficient UPEC strain is more sensitive to NO than a wild type strain [6,12]. The importance of NO-protection and flavohemoglobin for UPEC colonization has been addressed in previous studies. UPEC that had been pre-conditioned to nitrosative stress showed facilitated colonization of the mouse urinary tract [7] while an impaired colonization was observed following flavohemoglobin gene depletion [12]. Moreover, elevated *hmp* expression was found in UPEC isolates isolated from patients with UTI, suggesting that UPEC isolates face host-derived nitrosative stress during human UTI and activate the NO-detoxifying enzyme flavohemoglobin [12].

Pharmacological inhibition of flavohemoglobin might represent a new strategy to combat human infections, including UTI [10]. X-ray structural information has revealed that the flavohemoglobin protein possess large heme pockets capable of sequestering imidazole antibiotics [13]. Antifungal azoles, like miconazole, are able to inhibit the activity of microbial flavohemoglobin, including the NO dioxygenase activity of *E. coli*[14]. Furthermore, the binding of miconazole to the heme moiety of flavohemoglobin has been demonstrated to increase the intracellular oxidative stress and enhanced the antimicrobial activity against *Staphylococcus aureus*[15].

During the last decade an increasing prevalence of extended-spectrum β-lactamase (ESBL)-producing *E. coli* has been detected worldwide. Plasmid-mediated β-lactamase enzymes inactivate β-lactam antibiotics, which results in ineffective compounds and therapy failure. The majority of ESBL-producing bacteria are isolated from urine samples [16,17] and the prevalence of uropathogenic ESBL-producing isolates have increased in community-acquired UTIs the last decade [18]. The CTX-M-type β-lactamases are the dominant ESBLs and the CTX-M family is classified into five major groups based on similarities in their amino acid sequences [19]. In addition to resistance to most β-lactam antibiotics, multidrug resistance (i.e. resistance ≥ 3 different antimicrobial groups) is common [19]. Dissemination of multidrug-resistant ESBL-producing *E. coli* may in the future change uncomplicated, treatable urinary tract infections into life threatening infections and new therapeutic options are urgent. Microbial defence enzymes that enable the bacteria to resist host-derived factors have emerged as attractive targets for drug development [20]. Inhibition of factors involved in NO-defence may find applications as antimicrobial therapy by disable the bacterial resistance mechanisms and enhance the toxicity of host-derived or exogenously administered NO.

The aim of the present study was to investigate the antibacterial effects of NO in multidrug-resistant ESBL-producing isolates with special focus on inhibition of the NO-consuming enzyme flavohemoglobin.

## Methods

### Bacteria

Four ESBL-producing *E. coli* isolates, recovered from urine from standard patient care individuals with indwelling urinary catheters and symptoms of UTI, were obtained from the Department of Microbiology, Örebro University hospital, Sweden. The identity of the patients was anonymized and after that further analysis of the bacterial strains was performed. Antimicrobial susceptibility testing was performed as recommended by the Swedish Reference Group for Antibiotics (http://www.folkhalsomyndigheten.se/raf). The *bla*_CTX-M_ gene was detected using real time PCR and nucleotide sequencing as previously described [21]. The CTX-M types and the antibiotic susceptibility of the different ESBL-producing *E. coli* are shown in Table 1.

**Table 1:** Summary of the results from the antibiotic susceptibility testing and the molecular characterization of the producing CTX-M-types

**Isolate**	**CTX-M subgroup**	**CTX-M type**	**Antibiotic resistance**
ESBL 1	CTX-M-9	CTX-M-24	CTX, CAZ, CTB, TMP, CIP, MEL, GEN
ESBL 6	CTX-M-1	CTX-M-15	CTX, CAZ, CTB, TMP, CIP
ESBL 7	CTX-M-1	CTX-M-15	CTX, CAZ, TMP, CIP, MEL
ESBL 9	CTX-M-9	CTX-M-14	CTX, CAZ, TMP, CIP, MEL

*Abbreviations*: *CTX* cefotaxime, *CAZ* ceftazidime, *CTB* ceftibuten, *TMP* trimethoprim, *CIP* ciprofloxacin, *MEL* mecillinam, *GEN* gentamicin.

UPEC strain J96, a pyelonephritis isolate, and an *hmp*-deficient mutant of J96 (J96Δ*hmp*) were also used in the study. Deletion mutation of *hmp* was constructed using homologous recombination and the FLP recombinase as previously described [12]. There was no difference in the growth ability (measurement of OD_600_ for 24 hours) between the mutant strain (J96Δ*hmp*) and the wild type strain (J96wt) [12]. Bacteria were maintained on tryptic soy agar (TSA) (Becton Dickinson, Le Pont Claix, France).

### Antimicrobial agents

All antimicrobial solutions were prepared freshly immediately before use. DETA/NO belongs to the family of diazeniumdiolates that consists of a complex of NO bound to a polyamine parent compound that spontaneously decompose in a pH-dependent, first-order process [22]. Stock solutions of DETA/NO (DETA/NONOate, Alexis Biochemicals, Lausen, Schweiz) were prepared in PBS (Invitrogen; Grand Island N.Y., USA), cefotaxime and polymyxin B nonapeptid (PMBN; Sigma-Aldrich, St. Louis, USA) were prepared in sterile water. Nitrofurantoin (Sigma-Aldrich, St. Louis, USA) was prepared in DMSO (Merck, Hohenbrunn, Germany) and miconazole (Sigma-Aldrich, St. Louis, USA) was dissolved in 25% ethanol (70%) and 75% polyetylenglykol 400 while heating for 1 h at 60°C.

### Determination of MIC

MIC (minimum inhibitory concentration) was determined by the broth dilution test. The test substances were inoculated with a bacterial suspension (~ 10^6^ CFU/ml) in Luria-Bertani (LB) broth (Lennox, Becton Dickinson, Franklin Lakes, USA) for 8–24 h at 37°C. All MIC tests were repeated in two isolates and at least twice. The MIC value for cefotaxime was determined in a cephalosporin-sensitive uropathogenic *E. coli* isolate. The MIC value for DETA/NO was determined after 8 h since bacterial re-growth was seen in all tubes after 24 h. A working concentration of 4 × MIC was used in all experiments.

### Evaluation of bacterial viability

An overnight culture grown in LB broth, at 37°C on shake at 200 rpm, were diluted 1/1000 to give a bacterial count of approximately 10^6^ CFU/ml in the tube. Time-zero samples (starting inoculums) were taken and the number of viable colonies determined as described below. Bacterial cultures were combined with antibiotics or relevant vehicle and incubated in darkness at 37°C. Samples were taken at different times after addition of antibiotics or vehicle (2, 4, 8 and 24 h) depending on the experimental protocol. The samples were diluted in PBS and at least three serial dilutions were plated on TSA-plates. Following overnight culture at 37°C, bacterial CFU/ml was determined by using mean from two dilutions. Growth was calculated as the numbers of CFU/ml in treated cultures or controls divided by the number of CFU/ml formed upon the plating of the initial starting inoculums and expressed as log CFU/ml.

### Experimental protocol

1) The bacterial viability of DETA/NO (4 × MIC, 4 mM), cefotaxime (4 × MIC, 0.26 μg/ml) and nitrofurantoin (4 × MIC, 32 μg/ml) was compared in all four ESBL-producing isolates after 8 h of treatment. Two isolates (ESBL 1 and ESBL 7) were further analysed at 2 and 4 h of treatment. 2) To confirm that the effect of DETA/NO was caused by NO and not the parent compound, a tube with DETA/NO in solution was inactivated at 37°C for 96 h followed by exposure in open air for 20 h. 3) To examine the involvement of the NO-consuming enzyme flavohemoglobin, an *hmp*-deficient UPEC strain (J96Δ*hmp*) and the wild type J96 strain were exposed to DETA/NO for 4, 8 and 24 h and the viability evaluated. 4) The effect of the imidazole antibiotic miconazole (50 μM) in combination with DETA/NO was examined in ESBL isolate 7 at 4, 8 and 24 h. 5) PMBN (5 μg/ml), a compound that increases cell membrane permeability [23], was examined in combination with miconazole and DETA/NO and the viability evaluated in all four ESBL isolates and in J96Δ*hmp.*

### Statistical analysis

Data are expressed as mean ± SEM. Differences between groups were assessed by the unpaired two-tailed Student’s t-test. Results were considered statistically significant at p < 0.05. n = number of independent biological replicates.

## Results

### Characteristics of the isolates

Antibiotic susceptibility testing revealed that the four isolates were resistant to cefotaxime, ceftazidime, trimethoprim and ciprofloxacin. In addition, resistance to ceftibuten, mecillinam and gentamicin was detected in some isolates (Table 1). Nucleotide sequencing revealed that the included isolates were represented by the CTX-M types CTX-M-15, CTX-M-14 and CTX-M-24 (Table 1).

### The antibacterial effect of DETA/NO

**Figure 1:**
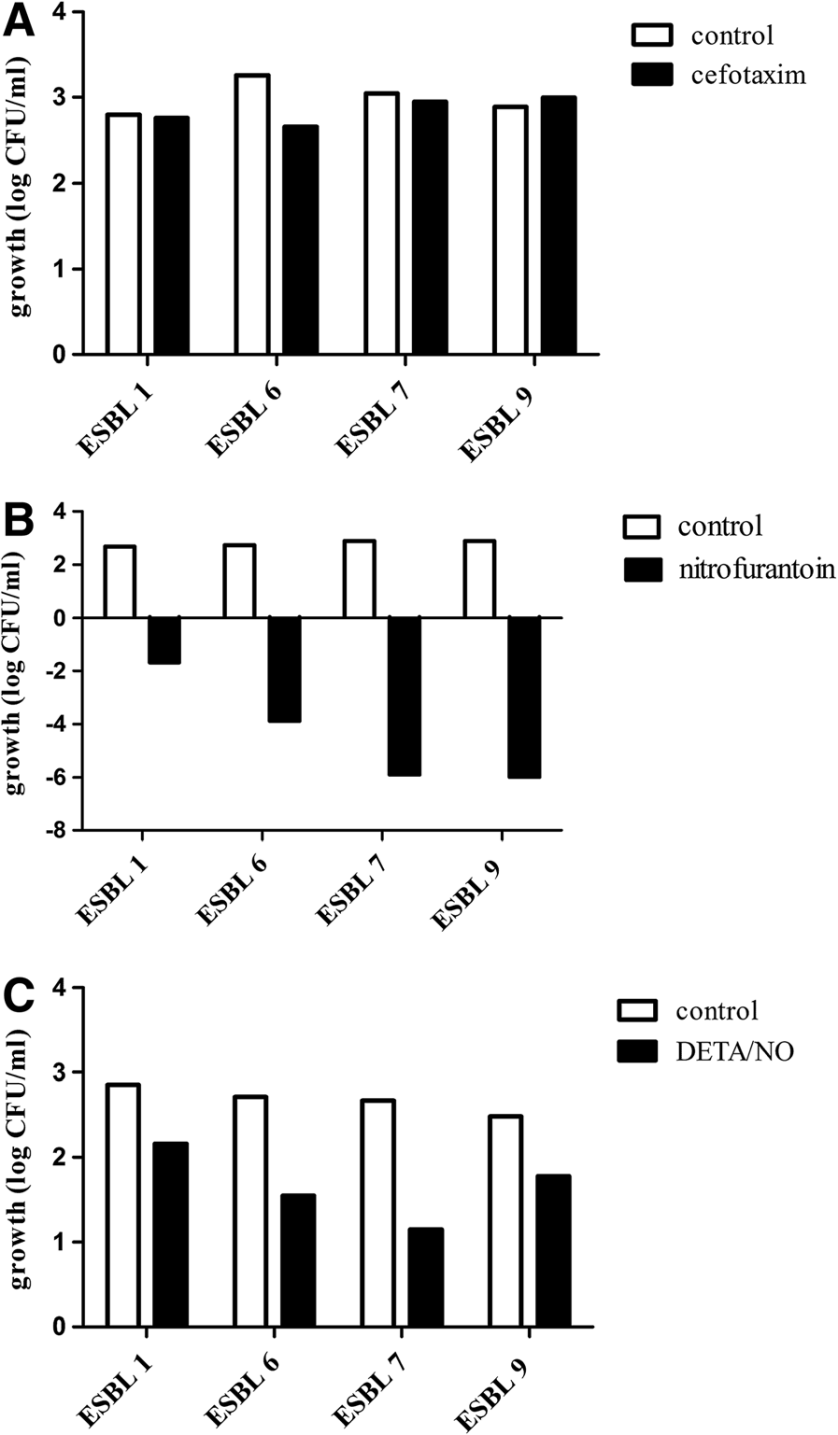
**A comparison of the antibacterial effect of cefotaxim, nitrofurantoin and DETA/NO.** The effect of cefotaxim **(A)**, nitrofurantoin **(B)** and DETA/NO **(C)** on bacterial viability studied in four different ESBL-producing isolates (ESBL 1, ESBL 6, ESBL 7, ESBL 9) after exposure for 8 h. Growth is shown as log CFU/ml of treated bacteria or controls (untreated) and compared to the initial starting inoculum. Representative data from one experiment is shown and similar results were obtained in at least two independent experiments for each isolate.

**Figure 2:**
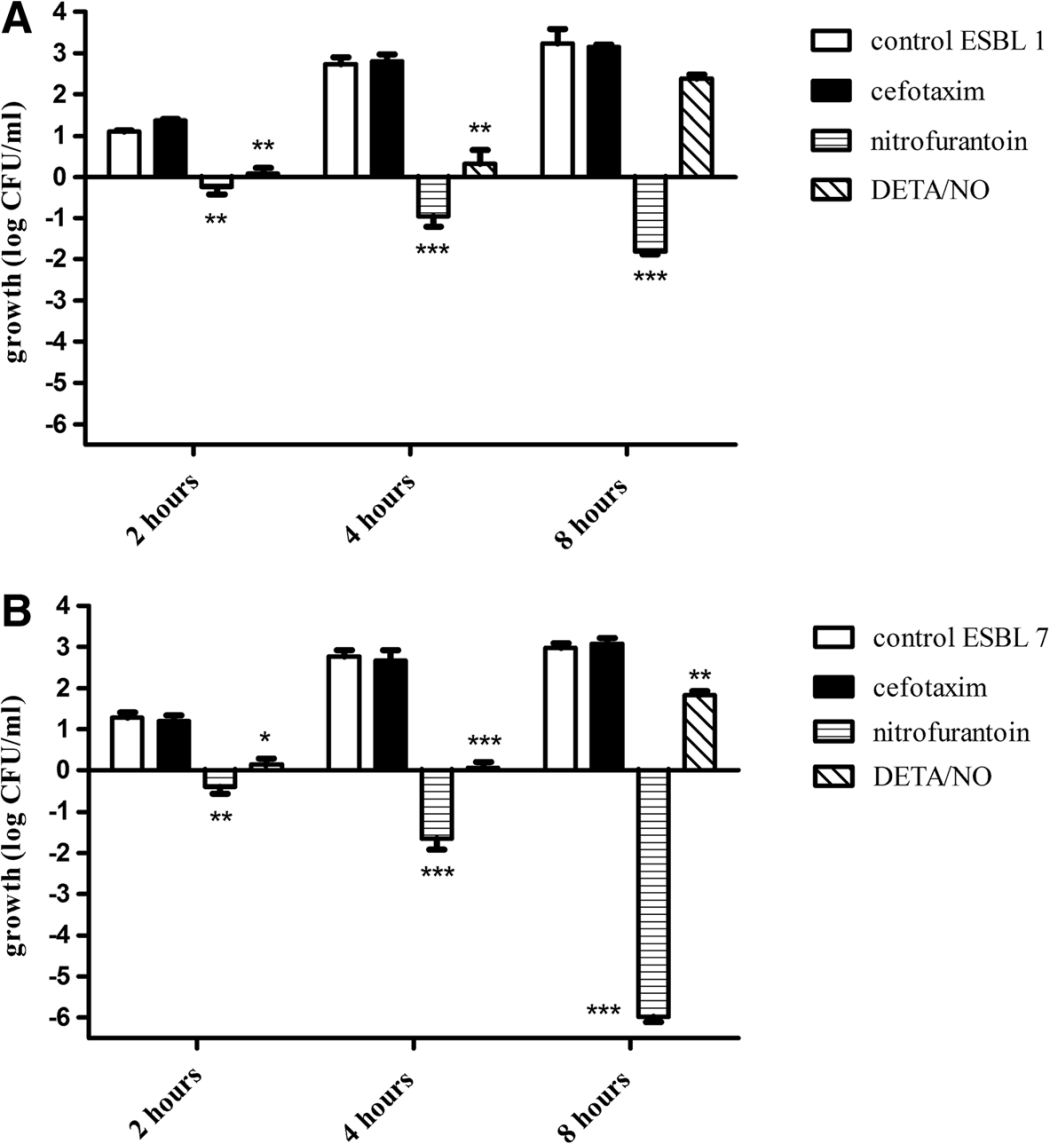
**Time-course studies of the antibacterial effect of cefotaxim, nitrofurantoin and DETA/NO.** Time-course study of cefotaxim, nitrofurantoin and DETA/NO on bacterial viability studied in **(A)** ESBL-producing isolate 1 and **(B)** ESBL-producing isolate 7 after exposure for 2, 4 and 8 h. Growth is shown as log CFU/ml of treated bacteria or controls (untreated) and compared to the initial starting inoculum. Data are expressed as mean ± SEM of three independent experiments. *p < 0.05, **p < 0.01, ***p < 0.001 vs control.

The antibacterial effect of the NO-donor DETA/NO on the ESBL-producing isolates was compared with the effect of the established antibiotics cefotaxime and nitrofurantoin after exposure for 8 h. Untreated isolates (controls) showed a growth of 2–3 log units during the 8 h period (Figure 1). As predicted, based on the antibiotic susceptibility tests, all four ESBL-producing isolates were resistant to the β-lactam antibiotic cefotaxime (Figure 1A), but sensitive to nitrofurantoin (Figure 1B). The growth response in DETA/NO-treated isolates was reduced by approximately 1–1.5 log units compared to controls (Figure 1C). The ESBL isolates 1 and 7, representing the most and the least DETA/NO-sensitive isolates were further evaluated by time-course studies after exposure to DETA/NO, cefotaxime and nitrofurantoin for 2, 4, and 8 h (Figure 2). In both isolates, DETA/NO demonstrated a significant growth inhibition compared to controls after 2 and 4 h of exposure (Figure 2). However, after 8 h the growth inhibitory effect was less pronounced and only significant in ESBL isolate 7 (Figure 2). A second dose of DETA/NO, administered after 4 h, did not prolong the growth inhibition at 8 h (data not shown). The effects of nitrofurantoin increased over time and, at least in ESBL isolate 7, the effect was bactericidal (decrease in viability by > 10^3^ log units) after 8 h. Both ESBL isolates were resistant to cefotaxime at all time points tested (Figure 2).

DETA/NO belongs to the family of diazeniumdiolates and is a complex of NO bound to a polyamine parent compound. An inactivated DETA/NO solution (prolonged heating and open air exposure) was found to lack antibacterial effects at 2, 4 and 8 h (data not shown), verifying that the antibacterial effect of DETA/NO is caused by NO.

### Inhibition of flavohemoglobin by gene deletion

**Figure 3:**
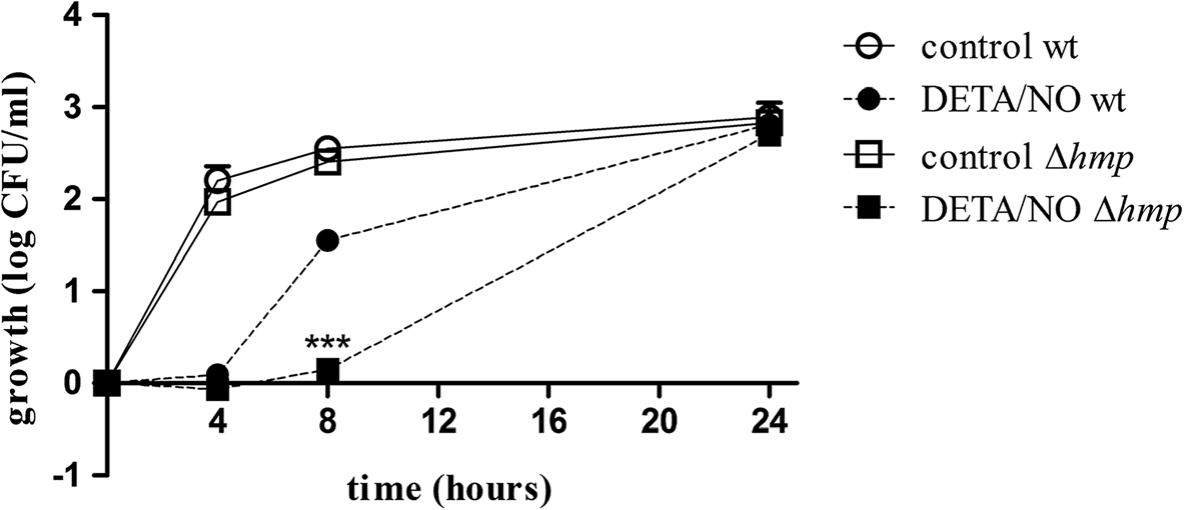
**The antibacterial effect of DETA/NO in a mutant strain lacking the flavohemoglobin gene (*hmp*).** The effect of DETA/NO on bacterial viability in an *hmp*-deficient UPEC strain (Δ*hmp*) and a wild type (wt) UPEC strain after exposure for 4, 8 and 24 h. Growth is shown as log CFU/ml of treated bacteria or controls (untreated) and compared to the initial starting inoculum. Data are expressed as mean ± SEM of three independent experiments. ***p < 0.001 DETA/NO wt vs DETA/NO Δ*hmp.*

To examine the role of flavohemoglobin for the antibacterial effect evoked by DETA/NO an *hmp*-deficient UPEC strain (J96Δ*hmp*) was used. The growth response of untreated J96Δ*hmp* and the wild type strain did not differ during the experimental period of 24 h (Figure 3). However, the J96Δ*hmp* strain showed a more prolonged growth inhibition in response to DETA/NO compared to wild type J96 (p < 0.001 at 8 h; Figure 3). The growth of the *hmp*-deficient strain recovered after 24 h (Figure 3).

### Pharmacological modulation of the antibacterial effect of DETA/NO

**Figure 4:**
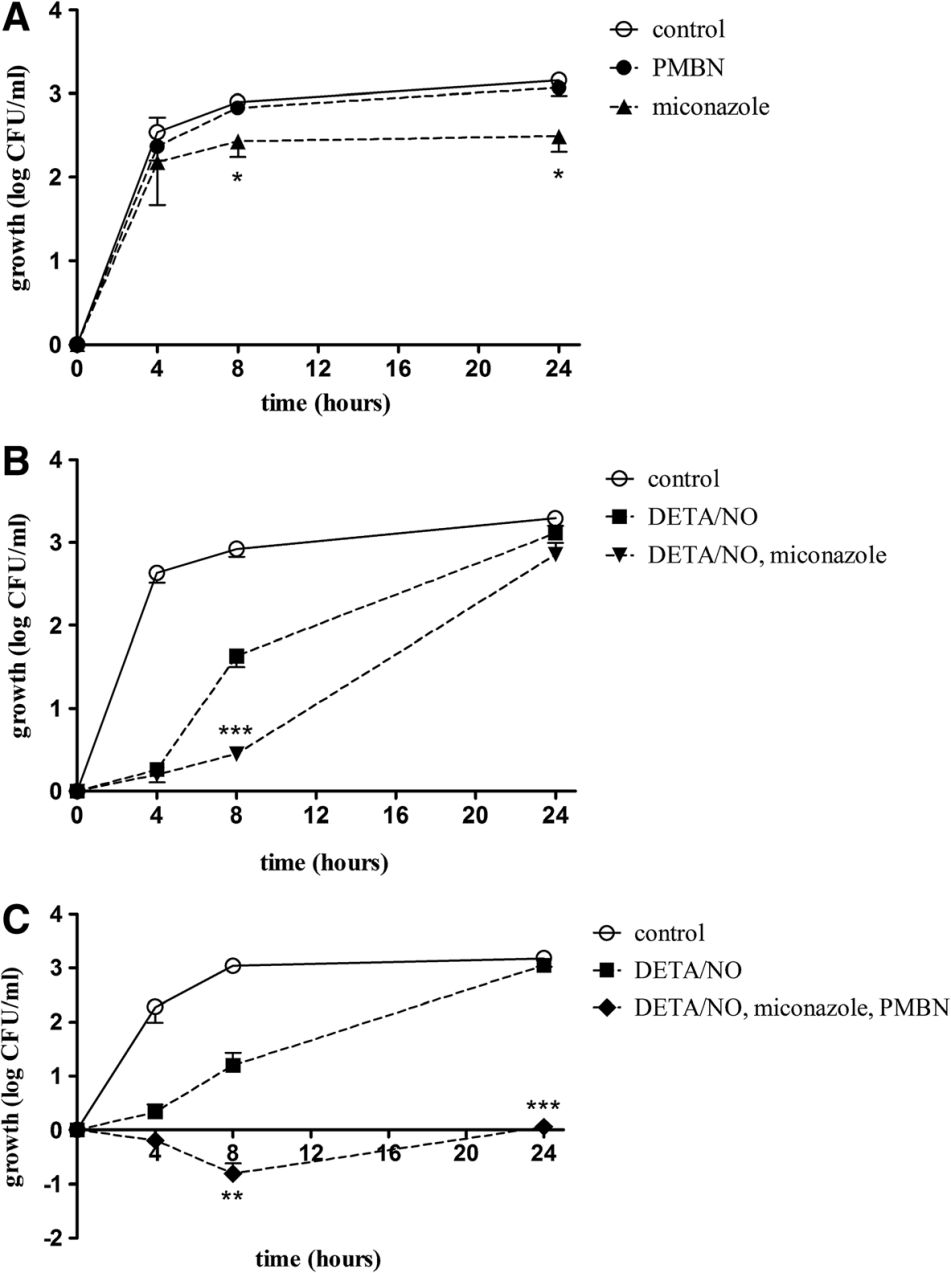
**The antibacterial effect of DETA/NO in combination with miconazole and polymyxin B nonapeptide.** The effect of DETA/NO on bacterial viability when combined with miconazole and polymyxin B nonapeptide (PMBN) in ESBL-producing isolate 7 after exposure for 4, 8 and 24 h. Growth is shown as log CFU/ml of treated bacteria or controls (untreated) and compared to the initial starting inoculum. **(A)** The effects of miconazole and PMBN *per se* are shown. *p < 0.05 control vs miconazole **(B)** The effects of DETA/NO alone and in combination with miconazole are shown. ***p < 0.001 DETA/NO vs DETA/NO + miconazole **(C)** The effects of DETA/NO alone and DETA/NO in combination with miconazole and PMBN are shown. **p <0.01, ***p < 0.001 DETA/NO vs DETA/NO + miconazole + PMBN. All data are expressed as mean ± SEM of three-four independent experiments.

The imidazole antimicrobial agent miconazole was used to examine whether pharmacological inhibition of flavohemoglobin affects the antibacterial effects of DETA/NO. Miconazole (50 μM) *per se* had small effects on the growth response when compared to controls (Figure 4A). When DETA/NO and miconazole were combined, the growth inhibition at 8 h was significantly more pronounced (p < 0.001) for the combination treatment than for DETA/NO alone (Figure 4B). However, after 24 h the differences in growth between the treatments were small and overall the viability was not significantly different from the untreated controls (Figure 4B).

**Figure 5:**
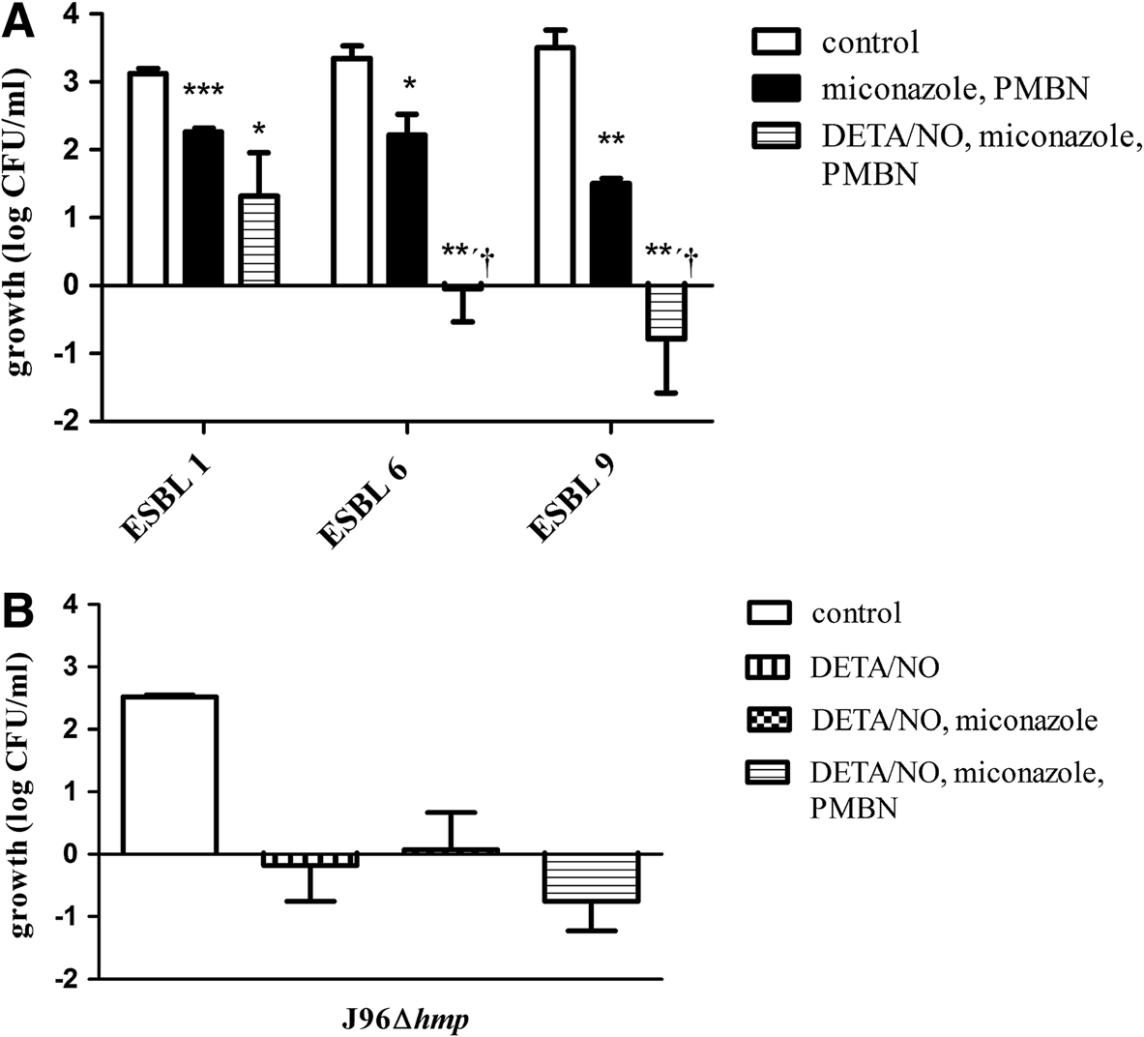
**The antibacterial effect of DETA/NO in combination with miconazole and polymyxin B nonapeptide in three additional ESBL-producing isolates and in a mutant strain lacking the flavohemoglobin gene (*hmp*). (A)** The effect of DETA/NO on bacterial viability when combined with miconazole and polymyxin B nonapeptide (PMBN) in three additional ESBL-producing isolates (ESBL 1, ESBL 6, ESBL 9) after exposure for 24 h. *p < 0.05, **p < 0.01, ***p < 0.001 vs control, †p <0.05 DETA/NO + miconazole + PMBN vs PMBN + miconazole. **(B)** The effect of DETA/NO on bacterial viability when combined with miconazole and polymyxin B nonapeptide (PMBN) in an *hmp*-deficient UPEC strain (Δ*hmp*) after exposure for 8 h. Growth is shown as log CFU/ml of treated bacteria or controls (untreated) and compared to the initial starting inoculum. Data are expressed as mean ± SEM of three independent experiments.

Hydrophobic antibiotics, like miconazole, have poor cell membrane permeability in gram-negative bacteria. Polymyxin B antibiotics, like polymyxin B nonapeptide (PMBN), can be used to increase the bacterial cell wall permeability of *E. coli*[23]*.* Time-course studies showed that PMBN (5 μg/ml) *per se* had no effect on the bacterial viability at 4, 8 or 24 h (Figure 4A). In MIC studies, PMBN had no effect on bacterial growth at the highest concentration tested (64 μg/ml). Miconazole and PMBN were used in concentrations that *per se* had no or minor effect on bacterial viability (Figure 4A). When DETA/NO was combined with miconazole and PMBN a marked and prolonged bacteriostasis for up to 24 h (p < 0.001) was noted in ESBL isolate 7 (Figure 4C). A prolonged bacteriostasis after treatment of DETA/NO in combination with miconazole and PMBN was also noted in ESBL isolates 6 (p < 0.01) and 9 (p < 0.01), while the effect was less pronounced in ESBL isolate 1 (p < 0.05; Figure 5A). PMBN and miconazole in combination slightly reduced the bacterial growth (Figure 5A). DETA/NO and PMBN in combination showed full recovery of growth after 24 h (data not shown).

### The effect of miconazole in the hmp-deficient UPEC strain

To investigate whether the effect of miconazole can be explained by inhibition of flavohemoglobin, miconazole was further evaluated in the *hmp*-deficient UPEC strain (J96Δ*hmp*). This strain does not express the flavohemoglobin protein and should therefore be insensitive to pharmacological inhibition of flavohemoglobin. As previously noted (Figure 3), DETA/NO inhibited the growth of the *hmp*-deficient UPEC during the 8 h study period. The addition of miconazole did not further enhance the growth inhibitory response evoked by DETA/NO (Figure 5B). The inhibitory effect of miconazole and DETA/NO in combination with PMBN was not significantly different from the effect by DETA/NO alone (Figure 5B).

## Discussion

The present study investigated the antibacterial effects of NO in multidrug-resistant ESBL-producing isolates with special focus on inhibition of the NO-consuming enzyme flavohemoglobin. Measuring bacterial sensitivity to gaseous NO is difficult because NO *per se* is stable for only minutes under physiological conditions [24]. Instead, NO-releasing compounds e.g., DETA/NO can be added to bacterial cultures to evaluate the growth response. In *E. coli*, NO acts in a bacteriostatic fashion and its targets include respiratory enzymes like cytochromes *bo* and *bd* and biosynthesis pathways of branched-chain amino acid [25]. In our study, DETA/NO induced a temporary growth inhibition in ESBL-producing UPEC isolates but after 8 hours of DETA/NO exposure a resumed growth was found. A second dose of DETA/NO, administered after 4 hours, did not prolong the growth inhibition (data not shown), suggesting that stress-response factors and NO-defence mechanisms may have been activated by the first dose. All isolates were resistant to cefotaxime but nitrofurantoin showed a time-dependent bactericidal effect. Nitrofurantoin is an antibiotic used for treatment of uncomplicated UTIs, and is so far effective against many isolates of ESBL-producing *E. coli*[26].

Upon diffusing into the bacteria, NO may react with Fe-S clusters, undergo autoxidation or be consumed directly through enzymatic detoxification [4]. Under aerobic conditions the vast majority of intracellular NO in *E. coli* is consumed through flavohemoglobin detoxification [4,9,10]. The flavohemoglobin enzyme is not constitutively expressed and needs to be induced by gene transcription [9,10]. We have previously shown that uropathogenic *E. coli* increase gene and protein expression of flavohemoglobin after exposure to DETA/NO [6,12]. Thus, induction of the flavohemoglobin enzyme and a fast consumption of NO to submicromolar intracellular NO concentrations may explain the temporary growth inhibition with a subsequent growth recovery in our experiments. Indeed, a mutant strain lacking the flavohemoglobin enzyme (J96Δ*hmp*) showed prolonged inhibition of growth, but after 24 hours this mutant also showed resumed growth. It has previously been verified that the *hmp*-deficient mutant used in the present study does not express the flavohemoglobin gene or protein when exposed to DETA/NO [12]. In the absence of functional flavohemoglobin NO is predicted to be metabolized mainly through autoxidation, enzymatic reduction by NorV and NrfA and by Fe-S nitrosylation [4]. Inhibition of flavohemoglobin by gene deletion was performed in a non ESBL-producing UPEC strain (J96). Gene deletion in ESBL-producing isolates is hampered by the obvious difficulties to find selection antibiotics in these multidrug-resistant isolates. However, we have confirmed a marked increase in *hmp* expression in an ESBL-producing isolate by real time RT-PCR when exposed to DETA/NO (data not shown), confirming that *hmp* is induced by DETA/NO also in ESBL-producing isolates.

Miconazole is known to interfere with synthesis of fungal and bacterial lipid membranes as it restrains the ergosterol synthesis [27], but recent studies also suggest that azole antibiotics target the flavohemoglobin enzyme [14,15]. Miconazole in combination with DETA/NO prolonged the DETA/NO-induced growth inhibition in ESBL-producing UPEC isolates. Notably, the pattern of DETA/NO-evoked growth inhibition achieved by addition of miconazole was similar to the pattern noted after *hmp*-deletion. Furthermore, the fact that miconazole did not increase the DETA/NO-induced growth inhibition in an *hmp*-mutant strain, support that inhibition of flavohemoglobin [14] contributes to the antibacterial effect of miconazole in our experiments. Thus, the prolonged growth inhibition evoked by DETA/NO and miconazole in combination may be a result of interactions of miconazole with flavohemoglobin, causing both inhibition of NO dioxygenase activity and oxidative stress following high levels of cytotoxic superoxide production [14,15]. In agreement with our results, intracellular survival studies in activated NO-producing macrophages demonstrated decreased survival of miconazole-treated *S. aureus* compared to untreated bacteria [15]. DETA/NO and miconazole have a synergistic antifungal effect in *Candida* species [28], and the present study demonstrates that these two compounds also caused an enhanced antibacterial effect against multidrug-resistant ESBL-producing *E. coli* isolates.

It is noteworthy that inhibition of flavohemoglobin activity by miconazole is more pronounced in purified enzyme than in intact *E. coli*[14], in line with the poor membrane permeability of *E. coli* to imidazole antibiotics [29]. Polymyxin B antibiotics may be used to sensitize the outer membrane of gram-negative bacteria to hydrophobic antibiotics [23]. We used polymyxin B nonapeptide (PMBN), a compound that increases the cell permeability in *E. coli* without affecting the bacterial viability [23,30], to avoid that polymyxin B mask the antibacterial effects of NO. PMBN *per se* had no antibacterial effect, while miconazole at the concentration used showed a minor inhibitory effect on UPEC growth. An *in vitro* synergism of miconazole and polymyxin B has been reported in *E. coli*, related predominantly to the ability of polymyxin B to increase the penetration of miconazole to the intracellular space [31]. In our experiments, miconazole and PMBN in combination caused a significant inhibition of growth compared to untreated controls. Interestingly, when DETA/NO was added to miconazole and PMBN a prolonged bacteriostatic response that persisted for 24 hours was observed. It is not likely that the underlying mechanism is a more effective inhibition of NO-detoxification by flavohemoglobin since the *hmp*-mutant showed recovered growth after 24 hours. However, a better access of miconazole to intracellular targets like flavohemoglobin, when combined with PMBN, may cause enhanced antibacterial activity through magnification of intracellular oxidative stress responses [15]. Furthermore, increased formation of toxic peroxynitrite (ONOO^−^), a potent oxidant formed from NO and superoxide radical [1], could conceivably contribute to the spectrum of potential antibacterial mechanisms of the combination treatment. The bacterial cell membrane is another possible target and miconazole is known to affect the integrity of the lipid membrane [27,32]. Morphological analysis have revealed cell membrane deteriorations with widespread structural deformations as a consequence of NO exposure in *E. coli*[33], and a synergistic effect of all three substances on the cell membrane is possible. However, the exact mechanisms for the prolonged bacteriostasis evoked by DETA/NO in combination with miconazole and PMBN need to be further studied. Interestingly, impaired adhesion to host renal epithelial cells and broken fimbriae has been reported after NO exposure in *E. coli*[33,34]. This suggests that the antibacterial effects of NO may be widespread and that NO not only has growth inhibitory effects but also may affect bacterial virulence properties and host activating mechanisms.

The ESBL-producing UPEC isolates used in the present study were obtained from patients with catheter-associated UTI. Indwelling medical devices, including urinary catheters and biofilm formation increase the risk of bacterial infection and result in considerable antimicrobial use. When these infections are caused by multidrug-resistant bacteria commonly used empirical antimicrobial therapy are not effective [35]. Administration of NO directly into the bladder through a silicone balloon catheter represents a local delivery system for NO-based therapy and has been suggested as one strategy to prevent catheter-associated infections [5]. Urinary catheters impregnated with NO have been shown to inhibit both biofilm formation and planktonic *E. coli* growth [36]. A limitation of the present study is that the number of clinical isolates used is small. However, three out of four isolates responded identical and with a prolonged bacteriostasis to the triple combination. These clinical isolates represented different CTX-M enzymes but it is, however, not possible to draw any conclusions on possible correlations between susceptibility to treatment and the CTX-M enzyme based on this material. Importantly, two of the isolates belonged to the CTX-M-15 ESBL type and the sequence type 131 (data not shown) which represent the dominating worldwide emerging CTX-M type and clone in community-acquired UTIs [37,38].

Colistin, a polymyxin antibiotic, has regained interest for its activity against multidrug-resistant gram negative pathogens, including those harbouring carbapenamases [39]. Thus, the emergency of multi-resistant pathogens encourages rediscovery of older antibiotics with activity against these resistant bacteria and in new combinations. Our data suggest that two existing antibiotics, an azole antifungal and a polymyxin B compound, are able to enhance the antimicrobial effects of exogenously administered NO. In contrast to antibiotics that are specific to one or few bacterial species, the antibacterial effects of NO are broad and both gram-positive and gram-negative pathogens, including antibiotic-resistant isolates show sensitivity [40,41]. Development of resistance mechanisms to NO may be limited by its multiple biochemical targets, but metabolic adaptions to nitrosative stress including induction of flavohemoglobin [10] and lactate dehydrogenase in *S. aureus*[42] decrease the antimicrobial action of NO. Therefore, as highlighted in the present study, a combination treatment where exogenous NO is combined with an inhibitor of NO-protective mechanisms appears to be an attractive approach to improve the antimicrobial effects of NO.

## Conclusion

This work shows that the antibacterial effect of NO against multidrug-resistant ESBL-producing uropathogenic *E. coli* is improved by combination with miconazole and polymyxin B nonapeptide.

## Competing interests

The authors declared that they have no competing interests.

## Authors’ contributions

Conception and design of the study: KP. Laboratory work: CSB, AK, MK, AÖ. Data analysis and interpretation: CSB, AK, MK, KP. Manuscript writing: CSB, BS, KP. All authors read and approved the final manuscript.
